# DAILY FACTORS ASSOCIATED WITH EMOTIONAL DYSREGULATION AMONG DEMENTIA CAREGIVERS

**DOI:** 10.1093/geroni/igad104.1329

**Published:** 2023-12-21

**Authors:** Frank Puga, Danny Wang, Natashia Bibriescas, Abigail Poe, Loreli Alvarez, Rita Jablonski, David Vance

**Affiliations:** University of Alabama at Birmingham, Birmingham, Alabama, United States; The Pennsylvania State University, University Park, Pennsylvania, United States; University of Texas at Austin, Austin, Texas, United States; University of Alabama at Birmingham, Birmingham, Alabama, United States; The University of Alabama at Birmingham, Birmingham, Alabama, United States; University of Alabama at Birmingham, Birmingham, Alabama, United States; University of Alabama at Birmingham, Birmingham, Alabama, United States

## Abstract

Family dementia caregivers experience increased burden and stress that can negatively impact emotion regulation and overall well-being. Further, a lack of social connection may contribute to emotion dysregulation among dementia caregivers. The purpose of this study was to examine relationships between daily caregiving stress, social isolation, emotion regulation, and depression in a sample of community-dwelling dementia caregivers. Participants (N=30) completed a baseline survey and a series of daily diaries over a 14-day period (n=323 data points) that asked about day-to-day caregiving experiences. Data were analyzed using mixed-level modeling. Higher than average stress related to behavioral symptoms of dementia was associated with an increase in the odds of experiencing feelings of losing control over one’s emotions (OR=1.08, 95% CI [1.01, 1.15], p<0.05). Similarly, higher perceived social isolation on a given day was associated with an increase in the daily odds of feeling a loss of control over one’s emotions (OR=1.28, 95% CI [1.00, 1.27], p<0.05). Further, feeling a loss of control over one’s emotions was associated with an increase in the daily odds of depression-related symptoms (OR=3.39, 95% CI [1.43, 8.08], p<0.01). These findings highlight the importance of addressing the emotional well-being of family dementia caregivers, particularly in the context of stress related to behavioral symptoms of dementia and social isolation. Caregiver support programs and interventions that focus on improving emotion regulation and reducing social isolation may help promote the mental health and well-being of dementia caregivers.

